# Alkylaminophenol and GPR17 Agonist for Glioblastoma Therapy: A Combinational Approach for Enhanced Cell Death Activity

**DOI:** 10.3390/cells10081975

**Published:** 2021-08-03

**Authors:** Phuong Doan, Phung Nguyen, Akshaya Murugesan, Nuno R. Candeias, Olli Yli-Harja, Meenakshisundaram Kandhavelu

**Affiliations:** 1 Molecular Signaling Group, Faculty of Medicine and Health Technology, Tampere University, P.O. Box 553, 33101 Tampere, Finland; phuong.doan@tuni.fi (P.D.); phunghatien.nguyen@tuni.fi (P.N.); akshaya.murugesan@tuni.fi (A.M.); 2 BioMediTech Institute and Faculty of Medicine and Health Technology, Tampere University, Arvo Ylpön katu 34, 33520 Tampere, Finland; olli.yli-harja@tuni.fi; 3 Science Center, Tampere University Hospital, Arvo Ylpön katu 34, 33520 Tampere, Finland; 4 Department of Biotechnology, Lady Doak College, Thallakulam, Madurai 625002, India; 5 Faculty of Engineering and Natural Sciences, Tampere University, P.O. Box 553, 33101 Tampere, Finland; ncandeias@ua.pt; 6 Computational Systems Biology Group, Faculty of Medicine and Health Technology, Tampere University, P.O. Box 553, 33101 Tampere, Finland; 7 Institute for Systems Biology, 1441N 34th Street, Seattle, WA 98103, USA

**Keywords:** glioblastoma, mesenchymal GBM, alkylaminophenol, GPR17 agonist, synergy, cell cycle arrest, intrinsic apoptotic pathway

## Abstract

Drug resistance and tumor heterogeneity limits the therapeutic efficacy in treating glioblastoma, an aggressive infiltrative type of brain tumor. GBM cells develops resistance against chemotherapeutic agent, temozolomide (TMZ), which leads to the failure in treatment strategies. This enduring challenge of GBM drug resistance could be rational by combinatorial targeted therapy. Here, we evaluated the combinatorial effect of phenolic compound (2-(3,4-dihydroquinolin-1(2H)-yl)(*p*-tolyl)methyl)phenol (THTMP), GPR17 agonist 2-({5-[3-(Morpholine-4-sulfonyl)phenyl]-4-[4-(trifluoromethoxy)phenyl]-4H-1,2,4-triazol-3-yl}sulfanyl)-N-[4-(propan-2-yl)phenyl]acetamide (T0510.3657 or T0) with the frontline drug, TMZ, on the inhibition of GBM cells. Mesenchymal cell lines derived from patients’ tumors, MMK1 and JK2 were treated with the combination of THTMP + T0, THTMP + TMZ and T0 + TMZ. Cellular migration, invasion and clonogenicity assays were performed to check the migratory behavior and the ability to form colony of GBM cells. Mitochondrial membrane permeability (MMP) assay and intracellular calcium, [Ca^2+^]i, assay was done to comprehend the mechanism of apoptosis. Role of apoptosis-related signaling molecules was analyzed in the induction of programmed cell death. In vivo validation in the xenograft models further validates the preclinical efficacy of the combinatorial drug. GBM cells exert better synergistic effect when exposed to the cytotoxic concentration of THTMP + T0, than other combinations. It also inhibited tumor cell proliferation, migration, invasion, colony-forming ability and cell cycle progression in S phase, better than the other combinations. Moreover, the combination of THTMP + T0 profoundly increased the [Ca^2+^]i, reactive oxygen species in a time-dependent manner, thus affecting MMP and leading to apoptosis. The activation of intrinsic apoptotic pathway was regulated by the expression of Bcl-2, cleaved caspases-3, cytochrome c, HSP27, cIAP-1, cIAP-2, p53, and XIAP. The combinatorial drug showed promising anti-tumor efficacy in GBM xenograft model by reducing the tumor volume, suggesting it as an alternative drug to TMZ. Our findings indicate the coordinated administration of THTMP + T0 as an efficient therapy for inhibiting GBM cell proliferation.

## 1. Introduction

Glioblastoma multiforme (GBM), a grade IV astrocytoma, is the most common malignant adult brain cancer [1]. Although combination therapy post-surgery has become the cornerstone for the anti-glioma treatment, patients have a dismal median survival of less than 15 months [2,3]. Treatment challenges exist for GBM, primarily due to the tumor heterogeneity, resistance to drug, blood–brain barrier, glioma stem cells, drug efflux pumps and DNA damage repair mechanisms [4].

Despite using TMZ as a first line drug in GMB therapy, its therapeutic effects are far reduced due to the enhanced activity of O^6^-methylguanine-DNA methyltransferase (MGMT). This DNA repair enzyme counteracts the TMZ induced DNA alkylation leading to the chemo-resistance against GBM treatment [5]. Although the classification of GBM into four distinct molecular subgroups such as proneural, neural, classical and mesenchymal subtypes [6] address the heterogeneity in GBM, intra-tumoral heterogeneity is the key determinant in therapy resistance leading to treatment failure [7]. These challenges lead us to understand how a conceivable therapeutic treatment could be best developed against GBM treatment.

Phenolic compounds are proven to be involved in various biological functions such as antioxidant, anti-inflammatory, chemo-preventive, and anticancer activity [8,9,10]. Several phenolic compounds such as vincristine [11], paclitaxel [12], omacetaxine [13] are successfully used as a chemotherapeutic agent against many forms of cancer. Earlier studies reported the role of phenolic compounds as an apoptotic inducer of GBM cells, with THTMP as the top potential compound exhibiting anticancer property [14]. Recently, we also identified that interaction of GPR17 with its ligand T0510-3657 (T0) could potentially regulate the GBM signaling communication and proliferation [15]. T0, a potential activator of GPR17 was found to exhibit better binding efficiency with stronger inhibitory activity than the known GPR17 agonist, MDL29951 [16]. RNA seq data of several GBM patient’s sample revealed the role of GPR17 in about 30 different crucial pathway interactions in the GBM signaling networks [15]. In addition, computational data analysis on the RNA-seq also have reported on the expression of GPR17 in 511 low-grade glioma (LGG) and 156 glioblastoma samples. Hence, we consider and evaluate the therapeutic effect of combination of THTMP and T0 with the TMZ, in GBM cells.

Several pre-clinical experiments have shown that the cytotoxic drugs against most of the cancers are effective to give synergism when given in combination. It is believed that a synergistic combination of different therapeutic agents against GBM cells could relapse the disease progression. Such combination therapies could be more efficient than monotherapy and chemotherapy against GBM. PTX combined with TMZ or cisplatin [17,18] showed an increased inhibitory effect against malignant GBM cells in-vitro. Notably, dual targeting of autophagic regulatory circuitry in gliomas using tricyclic antidepressants (TCAs) and P2Y12 inhibitors elicits the safe combination in treating glioma [19].

The rationale behind the combination therapy for treating GBM requires the identification of the best possible combination of the drug at effective doses that targets specific molecular mechanisms. Thus, the present study investigates the combinatorial administrations of a GPR17-ligand, phenolic compound with the chemotherapeutic agent. We evaluated the mechanistic effect and therapeutic potential of apoptosis induction in the mesenchymal GBM subtype using a combination of phenolic derivatives with GPR17 agonist and with a known anticancer agent, TMZ. We also identified the effect of combinatorial drug, THTMP + T0, in inducing cell death with higher cytotoxicity against GBM cell lines than TMZ through the activation of intrinsic apoptotic signaling pathways. We also assessed the anti-metastatic property of the combinatorial drug that inhibits the migration and invasion of the GBM cells. In-vivo preclinical validation also proves the potential of the drug in reducing the tumor volume in xenograft models, thereby suggesting its usability as a therapeutic agent against GBM treatment.

## 2. Materials and Methods

**Chemistry:** Preparation and spectral characterization of compound, THTMP was done as previously reported [20]. GPR17 agonist (T0510.3657 or T0) was purchased from AKos GmbH (Stuttgart, Germany).

**Cell culture:** Low-passage primary patients GBM cell lines, RN1, PB1, MMK1 and JK2 were procured from QIMR Berghofer, Medical Research Institute, Australia (gifted by Brett Stringer) and were approved by the human ethics committee of the Queensland Institute of Medical Research and Royal Brisbane and Women’s Hospital [21]. The cells were cultured in the serum-free RHB-A medium (Takara Clontech Cellartis, USA, Inc.) supplemented with growth factors like EGF and FGFb (Gibco, Grand Island, NY, USA), 0.1 mg/mL Streptomycin, 100 U/mL Penicillin (Sigma-Aldrich, St. Louis, MO, USA) in 1% Matrigel-coated flasks (Corning Life Science, St. Louis, MO, USA USA). The cells were maintained and cultured in the incubator at 37 °C in humidified air with 5% CO_2_ [22].

**Cytotoxicity assay:** RN1, PB1, MMK1 and JK2 cell lines were seeded in Matrigel-coated 12-well plates at a density of 1 × 10^5^ cells per well. Dose dependent analysis of THTMP and/or T0 and/or TMZ was performed with varying concentrations, such as 10 µM, 25 µM, 50 µM, 75 µM and 100 µM. The cells were incubated for 48 h at 37 °C and the cell viability was analyzed using trypan blue solution and Countess II FL Automated Cell Counter (ThermoFisher Scientific, Waltham, MA, USA). The percentage of growth inhibition was calculated relative to the DMSO-treated control wells. Dose responsive curve was calculated to identify the IC_50_. All the experiments were performed with three biological repeats and two technical repeats.

**Synergy screening assay:** Synergy screening assay was performed in MMK1 and JK2 cells with an initial density of 1 × 10^5^ cells per well. The cells (*n* = 6) were treated with combination of three-point dose series of THTMP (10 µM, 30 µM, 50 µM) and/or T0510.3657 (10 µM, 40 µM, 70 µM) with TMZ (10 µM, 50 µM, 100 µM). Cells were incubated in nine-point combination doses of either THTMP + TMZ, THTMP + T0 and/or T0 + TMZ for 48 h and cell viability was determined as described previously. The coefficient of drug interaction (CDI) was calculated from each combination using COMPUSYN method [23].

**Migration and invasion assay:** Migration and invasion assay were performed to assess the chemotactic capability and invasion of cells through the extracellular matrix. The assay was done in 6-transwell plates (*n* = 6) with the pore size of 8 µM (Corning Life Science, St. Louis, MO, USA). MMK1 and JK2 cells with the initial density of 1 × 10^5^ cells per well were seeded in 500 µL of fresh medium with THTMP (40 µM), T0 (40 µM) and THTMP + T0 (30 µM + 10 µM) and/or without compounds in the upper compartment. The lower compartment was filled with 1 mL of medium containing growth factors, such as EGF (20 ng/mL) and FGFb (10 ng/mL). The plates were then incubated at 37 °C with 5% CO_2_ for 18 h. For invasion assay, the upper compartment was coated with 100 µL of Matrigel (0.5 mg/mL) and the cells were seeded after 2 h. After 18 h of incubation, the membrane was fixed in ethanol and acetic acid (3:1) and further stained with 0.5% crystal violet. The cells which are not migrated/invaded were removed using a cotton swab. The well area was divided into 3 sections and multiple random fields of each section were chosen for counting the cells. The total number of the cells were counted at 40× magnification.

**Clonogenic assay:** Clonogenic assay was performed as described previously [24]. Briefly, the cells (*n* = 6) were incubated with THTMP (40 µM), T0 (40 µM) and THTMP + T0 (30 µM + 10 µM) for 48 h, 72 h, and 96 h. The cells were then harvested and plated in 6-well plate with the density of 5 × 10^5^ cells per well without Matrigel coating and incubated at 37 °C with 5% CO_2_ for 14 days. Later, the cells were fixed in ice-cold ethanol and acetic acid (3:1) for 10 min. The colonies were stained with 0.5% crystal violet prior to counting. Six fields of the well were taken randomly and used for counting. The colonies smaller than 30 µM were not accounted.

**Cell cycle assay:** The cells (*n* = 6) were treated with THTMP (40 µM), T0 (40 µM) and THTMP + T0 (30 µM + 10 µM) for 48 h and fixed in 70% ice-cold ethanol at 4 °C for 1 h. The cells were then washed in PBS and resuspended in 500 µL of PBS containing 2 µg/mL Propidium Iodide (Sigma-Aldrich, St. Louis, MO, USA), 0.2 mg/mL RNase and 0.1% triton X-100 and incubated at 37 °C for 30 min. The cells were maintained on ice before the image analysis using EVOS imaging system (ThermoFisher Scientific, Waltham, MA, USA). Images were analyzed using CellProfiler ver. 3.1.9 and Matlab ver. R2018b.

**Apoptosis assay:** MMK1 and JK2 cells at the initial density of 5 × 10^5^ were seeded per well in 6-well plates and treated with the THTMP and T0 single dosage and combination of THTMP + T0. Dead Cell Apoptosis Kit with Annexin-V/FITC and PI (ThermoFisher Scientific, Waltham, MA, USA) was used to analyze the apoptosis induction following the manufacturer’s protocol. The treated cells were incubated at RT for 15 min prior to the fluorescence measurements. The image acquisition was done using EVOS imaging system (ThermoFisher Scientific, Waltham, MA, USA) with 20× objective magnification.

**ROS assay:** MMK1 and JK2 cells were seeded in 12-well plates with the initial density of 1 × 10^5^ cells per well. Cells were allowed to grow overnight at appropriate cell culture condition. The cells were treated with THTMP, T0 and combination of THTMP + T0 for 5 h. Cells were harvested and incubated with 2 μM 2′,7′-dichlorodihydrofluorescein diacetate (H2DCFDA, Sigma-Aldrich), for 30 min at 37 °C with 5% CO_2_. Cultures were washed with PBS and recovered in pre-warmed medium for 20 min. Plate reader (Fluoroskan Ascent FL, Thermo Labsystems, Männedorf, Switzerland) was used to measure the fluorescence with the excitation at 485 nm and emission at 538 nm. Hydrogen peroxide (200 µM) (Sigma-Aldrich, St. Louis, MO, USA) was used as the positive control. The fold increase in ROS production was calculated based on the fluorescence intensity of treated, untreated, and blank samples.

**Calcium assay:** Cells were seeded in 96-well plates with the initial density of 1 × 10^4^ cells per well. At 60–70% confluency, the cells were incubated with 5 µM Fura-2 AM (Sigma-Aldrich, St. Louis, MO, USA) for 30 min at 37 °C and washed with PBS twice before the addition of 50 µL of medium. Fluorescent signal was measured every 5 min using a microplate reader (Spark^®^, Tecan, Thermo Labsystems, Männedorf, Switzerland) at two dual excitation/emission wavelengths 340/510 nm and 380/310 nm. After 10 min of measurement, 50 μL of PBS containing IC_50_ concentration of the tested compounds (THTMP, T0 and THTMP + T0) were added to the wells. Fluorescence measurement was carried out further for 1 h 30 min. The ratio of the fluorescence at the dual wavelengths (340 nm/380 nm) was used to calculate the changes in [Ca^2+^]i as previously described [25].

**Mitochondrial membrane potential assay:** The mitochondrial-specific cationic dye JC-1 (Thermo Fisher Scientific, Waltham, MA, USA) was used to measure the mitochondrial membrane potential [26]. The assay was performed with the same experimental protocol as described above for calcium assay except, the addition of 10 µg/mL of JC-1 instead of Fura-2 AM. Fluorescence intensity was measured using microplate reader (Spark^®^, Tecan, Thermo Labsystems, Männedorf, Switzerland) at an excitation and emission wavelength of 485 nm/530 nm and 535 nm/590 nm. The change in mitochondrial membrane potential was calculated based on the ratio at 590 nm to 530 nm.

**Expression profiling of apoptosis array:** A proteome profile of human apoptosis array was done (R&D systems, Minneapolis, MN, USA) following the manufacturer’s instruction. The array can capture 35 different apoptosis antibodies in duplicate on nitrocellulose membrane. MMK1 and JK2 cells at a density of 1 × 10^7^ cells/mL were treated with THTMP (30 µM) and T0510.3657 (10 µM) and DMSO as the control for 48 h. The procedure was performed according to the manufacturer’s protocol. Briefly, the cell lysates along with the cocktail of biotinylated detection antibodies were incubated with Proteome Profiler Human apoptosis array. Streptavidin-HRP and chemiluminescent detection reagents are added which produce the signals at each spot that correspond to the amount of phosphorylated protein. Images were captured using XENOGEN (Vivo Vision IVIS Lumina, Männedorf, Switzerland). The data were analyzed using ImageJ software.

**In-vivo anticancer studies:** In vivo anti-cancer activity was evaluated against glioblastoma U373-MG Uppsala in human tumor xenograft mice model. This was originally established at the University of Uppsala (https://web.expasy.org/cellosaurus/CVCL_2818, accessed on 24 June 2021) [27]. The protocols were approved by Institutional Animal Ethics Committee, ACTREC, Tata Memorial Centre, Navi Mumbai (Ethical number: 01/2015), adhering to CPCSEA guidelines (Registration Number: 65/GO/ReBiBt/S/99/CPCSEA). In-house bred six- to eight-week-old female mice were used in the experiments. Animals were maintained with utmost human care to minimize animal suffering before and during the experiments.

**Toxicity studies:** Intraperitoneal injection of THTMP + T0 was done in the immunocompetent Nod/Scid mice. Toxicity criteria was considered with the mortality and weight loss of ≥4 g/mouse. The dosage of the drug used was 20 mg/kg body weight of the animal and injected at every 7 days of interval for 36 days. The animals were continuously monitored for any mortality after post-dosing of drugs.

**Experimental design:** Human Tumor Xenograft-U-373MG Uppsala was developed in Nod/Scid female mice. Desired experimental groups of *n* = 6/group was maintained. The experimental group consist of control (Group A), vehicle control–DMSO (Group B), Positive control–TMZ (Group C) and THTMP + T0 (Group D). Tumor growth and tumor volume was measured using digital Vernier caliper (Pro-Max, Electronic Digital Caliper, Fowler-NSK, USA). Body weight, tumor volume and mortality of the mice was continuously monitored throughout the experimentation period of 36 days. After the experimentation period, the animals were sacrificed following the Institutional Animal Ethics Committee by injecting Pentobarbital 45 mg/kg, intra peritoneally.

***Statistical analysis:*** Relative tumor volume (RTV in cc), T/C (ratio of test versus control) and survival was calculated using the following formula. Tumor volume = [(w1 × w1 × w2) × (π/6)], where w1 and w2 were the smallest and the largest tumor diameter (cm), respectively. RTV was measured as tumor volume on the day of measurement/tumor volume on day 1. Antitumor effectiveness was indicated as T/C ratio.

The percentage treatment/control (T/C%) values and percent tumor regression values were calculated as follows:

(1) $$\;\;Relative\;Tumor\;Volume\;\left(RTV\right)\frac{T}{C}\;\left(\%\right)=\frac{RTV_{Test}}{RTV_{Control}}$$

(2) $$Tumor\;Regression\;\left(\%\right)=100-\left(\frac{T}{C}\times 100\right)$$

*RTV* = mean tumor volume of the drug-treated group on the study day of interest–mean tumor volume of the drug-treated group on the initial day of dosing; *C* = mean tumor volume of the control group. Biological activity was considered significant when *T/C* values were ≤0.42, as per the NCI, USA guidelines.

## 3. Results

### 3.1. Synergic Effect of THTMP + T0 against Patient Derived GBM Cells

**Figure 1:**
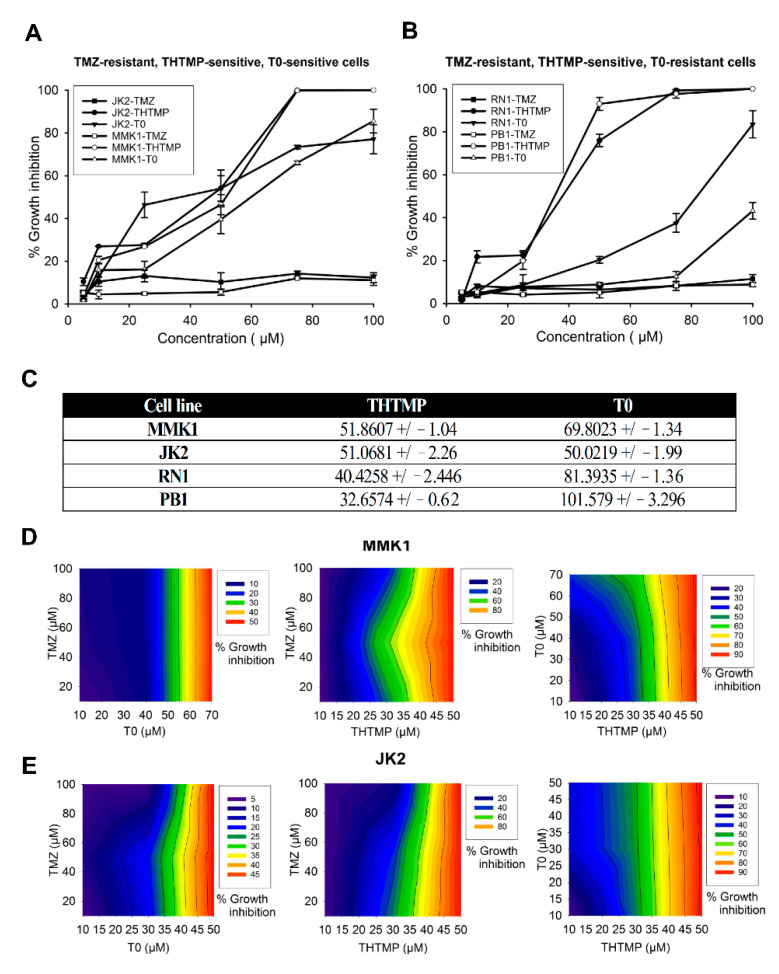
Effect of single and combination treatment of alkylaminophenol (THTMP) and GPR17 agonist (T0) on cell growth inhibition in multiple GBM patient tumor tissue-derived cells. The characteristics of TMZ-resistant, THTMP-sensitive, T0 sensitive cells (**A**), TMZ-resistant, THTMP-sensitive, T0-resistant cells (**B**) were observed at 48 h post-treatment. The IC_50_ values of THTMP, T0 for multiple GBM patient tumor tissue-derived cells (**C**). Cells treated with different combinatorial conditions, TMZ + T0, TMZ + THTMP, and THTMP + T0 with a series of concentrations (10 µM, 25 µM, 50 µM, 75 µM and 100 µM) on MMK1 (**D**) and JK2 (**E**). The data were shown as means ± SD, with *n* = 5.

Considering the inter-tumor heterogeneity of the GBM that classifies them into molecular subtypes, patient-derived cell lines such as MMK1, JK2, RN1 and PB1 [28] were used for the analysis of cell growth inhibition. The cells were treated with varying concentrations of TMZ, THTMP and T0 as described in the method section. We observed two distinct responses including TMZ-resistant, THTMP-sensitive, T0-senstivecells (Figure 1A) and TMZ-resistant, THTMP-sensitive, T0-resistant cells (Figure 1B).

At 48 h post-treatment, TMZ single treatment was not effective in reducing the cell viability in all the cell lines. It showed only 12% growth inhibition even at 100 µM and hence was denoted as TMZ resistant. Upon THTMP treatment, the cells which showed higher growth inhibition of about 90% at 75 µM were denoted as THTMP-sensitive. Likewise, PB1 and RN1 were classified as T0 resistant cell, since they showed only 12.5% and 37.5% of growth inhibition at 75 µM, whereas MMK1 and JK2 were considered as T0 sensitive cells due to their ability to moderately reduce the cell viability up to 73.4% at 75 µM (Figure 1A,B). The half maximal inhibitory concentration (IC_50_) deduced from the dose-responsive curve was presented in Figure 1C. From the above observation, it was noted that MMK1 and JK2 cells were sensitive to THTMP and T0 and hence selected for further analysis.

Synergistic effects of THTMP, T0 and TMZ were investigated upon combinatorial assessment of TMZ + THTMP, TMZ + T0 and THTMP + T0 using their respective IC_50_ for the selected cell lines. It is discernible that the combination of TMZ + T0 at 70 µM and 100 µM concentration does not show more than 50% inhibitory effect in both MMK1 and JK2 cells. On treatment with TMZ + THTMP, we observed an increased cell death to about 80% with significant percentage of growth inhibition of about 90% on treatment with THTMP + T0 at 50 µM/10 µM concentration. The gradient color in the graph (blue to red) shows the difference of the drug when given in combination. The color change was found to be possible only when there is an effect of combining drugs in a single effect. In addition, it is possible to observe from the graph that the % of growth inhibition at minimal concentration was lower for either drug, while we observed a promising effect at higher concentration (Figure 1D,E).

### 3.2. Evaluation of Drug Synergy by Combination Therapy

**Figure 2:**
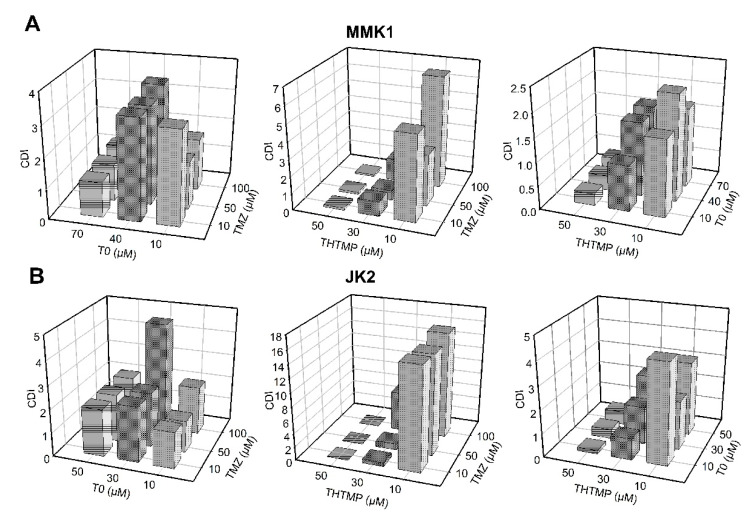
The coefficient of drug interaction (CDI) values for combinatorial drugs (T0 + TMZ), (THTMP + TMZ) and (THTMP + T0) of (**A**) MMK1 and (**B**) JK2 cells.

Drug combination effect of THTMP and T0 with TMZ was assessed by median effect analysis through the quantification of coefficient of drug interaction (CDI) using COMPUSYN method [23]. The (CDI) method has been used for the evaluation of the interaction combination effects in different ratios and concentrations [29,30,31]. To better understand and correlate the effect of combination of drugs on the growth inhibition, synergistic effect was calculated based on CDI value. The CDI was calculated based on the conservation assumption of drug interaction, and if CDI = 1 it was denoted as additivity, CDI < 1 as synergism and CDI > 1 as antagonism. The synergistic effect was best observed in THTMP + T0 combination, exhibiting lower CDI value than the THTMP + TMZ drug combination in both MMK1 and JK2 cells (Figure 2A,B). There was a negligible level of synergism in the TMZ + T0 combination. The increasing concentration of THTMP was found to be directly proportional to the increased synergistic effect, whereas T0 does not show significant differences in the CDI even at higher concentration. Although the ratio of THTMP:T0 corresponding to 50 µM:10 µM had significant synergic effect with 90% growth inhibition, this particular concentration causes the cells to float and makes further analysis difficult. Hence, 30 µM: 10 µM (CDI value 0.9186 for JK2 and 0.97967 for MMK1) concentration was chosen for further studies, as it also exhibited synergism with approximately 50% cell growth inhibition.

### 3.3. Combinatorial Drug THTMP + T0 Attenuates Migration and Invasion of GBM Cells

**Figure 3:**
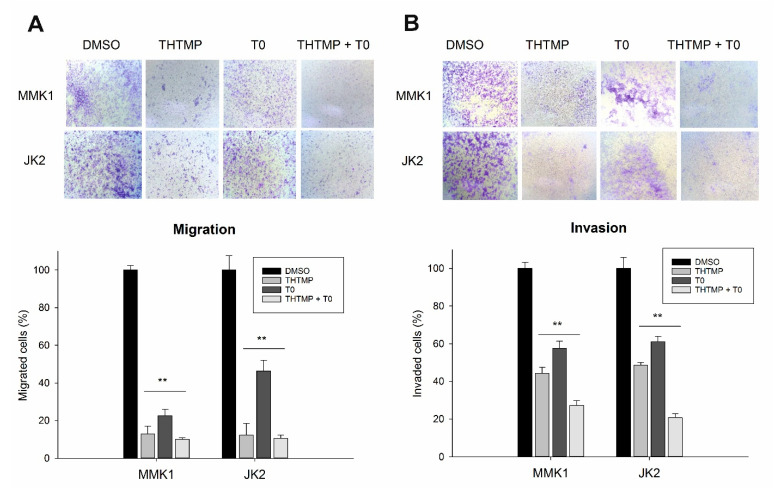
Analysis of cell migration and invasion assay. Effect of DMSO (control), THTMP, T0 and combination of THTMP + T0 on cell migration (**A**), invasion (**B**) on MMK1 and JK2 using transwell method. Microscopic images are the representation of the % of migration and invaded cells captured in 40× magnification. The data were shown as mean ± SD, *n* = 3, ** *p* < 0.01.

The decisive feature of metastasis is the migration and invasion of the cancer cells causing the disease progression. We investigated the anti-metastatic properties for the combinatorial drug, THTMP + T0 by assessing its ability to inhibit migration and invasion of GBM cells using transwell method. THTMP and T0 single treatment significantly inhibited the migration ability of MMK1 (22.5% and 12.9%) and JK2 (46.3% and 12.4%), with further decrease in the migration of less than 10% for the THTMP + T0 combination. It was also noted that THTMP has a better effect on MMK1 cell migration, whereas T0 affects the migration of JK2 cells more significantly than MMK1 cells (Figure 3A). A similar pattern was also observed in the invasion assay. THTMP and T0 single treatment reduced the invasion of cells to 44.3% and 57.5% in MMK1 and 61.1% and 48.7% of invaded cells in JK2, respectively. The combined effect of THTMP + T0 decreased the invasion of cells significantly to 27.2% and 20.7% in MMK1 and JK2 cells, respectively (Figure 3B). As a note, TMZ does not inhibit the migration and invasion of MMK1 and JK2 cells. Thus, the synergistic effect of THTMP + T0 facilitates the inhibition of migration and invasion activity in both the GBM cells.

### 3.4. Clonogenicity of THTMP + T0 in Primary GBM Tumor Cells

**Figure 4:**
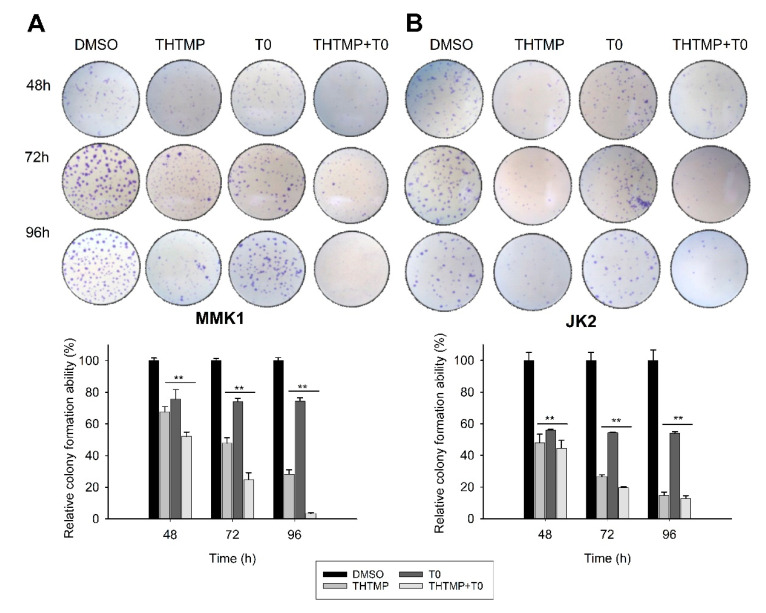
Effect of THTMP, T0 and combination of THTMP + T0 on colony formation ability on (**A**) MMK1 and **(B**) JK2. Time-lapse microscopic images of GBM cells upon treatment at 48 h, 72 h, and 96 h. The data were shown as mean ± SD, *n* = 3, ** *p* < 0.01.

We further performed the time-series clonogenic assay to analyze the efficacy of combinatorial drug THTMP + T0 in inducing the cell reproductive death by DNA damage. MMK1 and JK2 cells presented 47.9% and 55.5% reduction in the colony forming efficiency after the combinatorial treatment at 48 h, when compared with the control, DMSO. As the time increases, the effect of THTMP + T0 significantly reduced the number of cells to about 90% at 96 h of treatment. Although THTMP and T0 single treatment reduced the number of colony-forming cells, THTMP showed higher effect with 32.3% reduction on MMK1 cells when compared with T0 which showed only 24% reduction at 48 h post-treatment (Figure 4A). An analogous result was also observed for JK2 cells with 52.0% and 44.1% of colony reduction upon THTMP and T0 treatment, respectively (Figure 4B). These data strongly suggest that the combination of THTMP + T0 significantly inhibits the clonogenic potential of patients’ derived GBM cells.

### 3.5. THTMP + T0 Unveils the Cell Cycle Checkpoints in GBM

**Figure 5:**
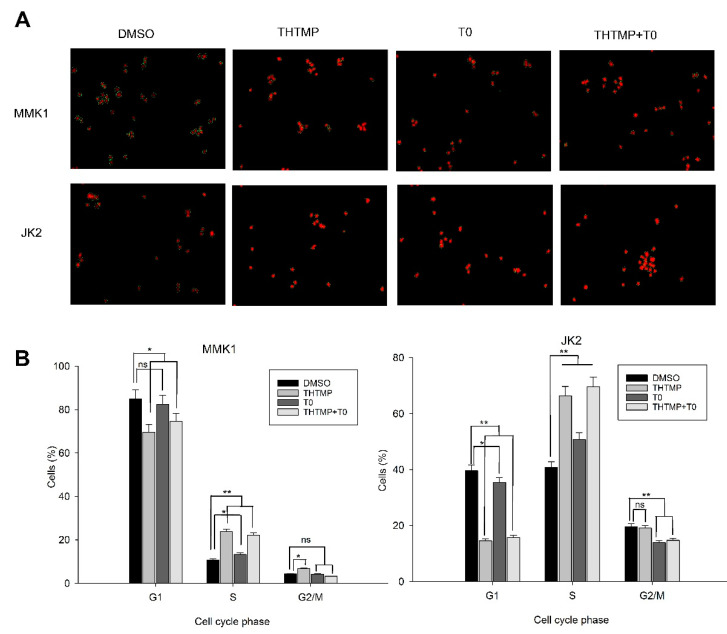
Effect of combinatorial drug on cell cycle analysis. THTMP and/or T0 compounds induced cell cycle arrest at S phase. Representative images of cell cycle analysis in which red color indicates DNA content of cells and green color indicates the area of cell segmentation (**A**). Percentage of cells in different cell cycle phases upon the treatment in MMK1 and JK2 cell lines (**B**). The data were shown as mean ± SD, *n* = 3, * *p* < 0.05, ** *p* < 0.01, ns = non-significance.

Dysregulation of cell cycle leading to uncontrolled cell division is one of the major characteristic features of tumor cells and thus arresting the cell cycle is considered an important mechanism for anti-glioma drugs. It was evident from the earlier experiments that THTMP + T0 induces cell death significantly, which prompted us to determine its effect on the cell cycle. Cell cycle analysis was performed using PI staining and the analysis of the fluorescence images was done using CellProfiler ver 3.1.9 (Figure 5A).

Upon THTMP + T0 treatment, the percentage of cells entering from G_2_ to M phase did not show significant differences in both cell lines. The shifting of MMK1 cells from G_2_ to M phase was 3.5% to 6.5%, whereas JK2 cells shifted from 14.0% to 19.3% (Figure 5B), with the corresponding increase in the percentage of cells in S phase than the control. It was also observed that approximately 10% of cells were seen in the S phase of DMSO-treated MMK1 cells with 23.7%, 13.5% and 22.10% of cells in THTMP, T0 and THTMP + T0 treated cells, respectively. Similarly, 40.8% of JK2 cells were seen at S phase in the DMSO treated cells, while 66.4%, 50.7% and 69.6% were noted in THTMP, T0 and THTMP + T0 treatment, respectively. Image analysis on the DNA content in both the cells treated with THTMP and/or T0 revealed the cell cycle arrest in S phase. This complements the data observed in the cell death analysis where a higher percentage of cells was observed in the S phase upon THTMP and THTMP + T0 treated cells when compared to T0 treated cells.

### 3.6. THTMP + T0 Activates Intrinsic Pathways of Apoptosis Induction

**Figure 6:**
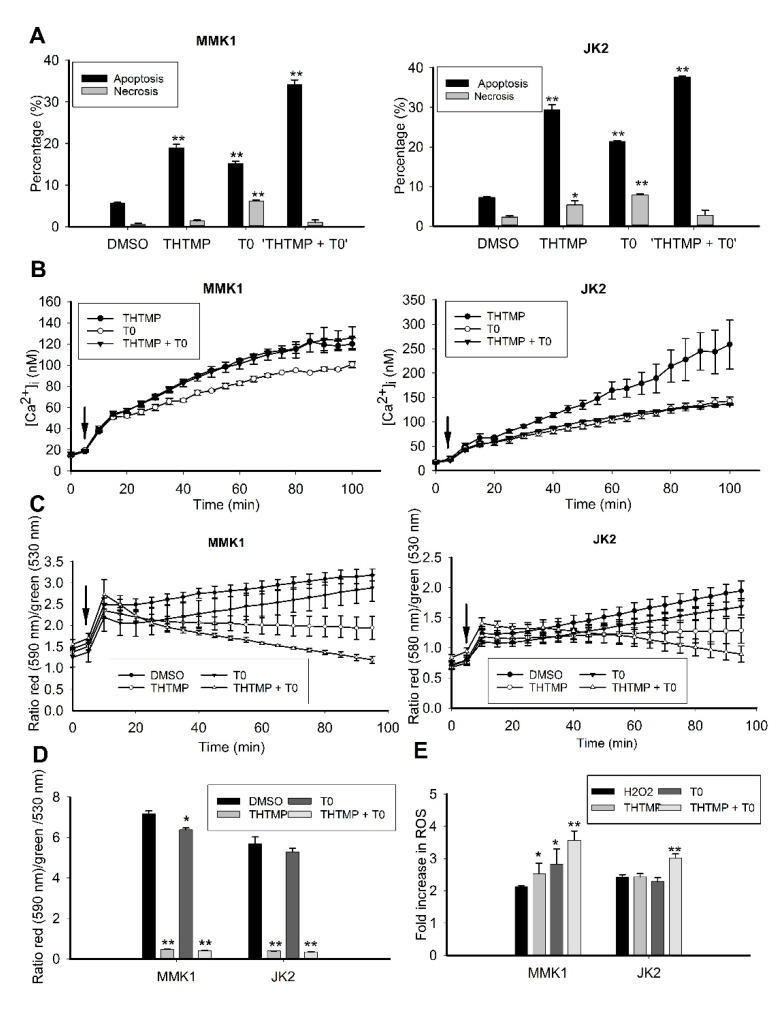
Cell death induction by THTMP, T0 and combination of THTMP + T0 on MMK1 and JK2 cells. Analysis of apoptosis induction in MMK1 and JK2 cells upon THTMP, T0 and THTMP + T0 (**A**). Time course measurement of [Ca^2+^]i using fluorescence spectrophotometry from Fura-2 loaded cells. Arrows indicate the time of the addition of the compounds (**B**). Change in mitochondrial membrane potential in MMK1 and JK2 cells within 100 min (**C**) and 24 h of treatment (**D**). Arrows indicate the time of the addition of the compounds. Fold change in ROS production (**E**). The data were shown as mean ± SD, *n* = 3, * *p* < 0.05, ** *p* < 0.01.

More detailed analysis on the effect of combinatorial drug, THTMP + T0 on cell death was evaluated by assessing the rate of apoptosis, intracellular calcium level, change in mitochondrial membrane potential and ROS production. The GBM cells were treated with the combinatorial drug, and the percentage of apoptotic and necrotic cells were determined using Annexin V and propidium iodide staining. Consistent with the cytotoxicity assay, the highest percentage of apoptosis was achieved in response to THTMP + T0 treatment, followed by THTMP and T0. The rate of apoptosis was found to be 34.2%, 18.9% and 15.2% in MMK1 cells, whereas JK2 showed 37.6%, 29.3% and 21.3%, respectively. The very least percentage of necrotic cells was observed in response to T0 treatment with 6.1% and 7.9% in MMK1 and JK2 cells, respectively (Figure 6A).

Ca^2+^ signaling is known to be critically involved in effectuation of the cell death. The key events in the apoptosis are triggered by the intracellular Ca^2+^ signals and hence the estimation of [Ca^2+^]i was performed upon induction by THTMP, T0, THTMP + T0 using Fura-2 assay. We observed a sustained increase in the [Ca^2+]^i in a time-dependent manner in both cell lines (Figure 6B). In MMK1 cells, [Ca^2+^]i signaling was increased till 100 min on treatment with either THTMP and THTMP + T0 than T0 treatment. JK2 cells showed higher [Ca^2+^]i signaling upon THTMP treatment than T0 and THTMP + T0 treatment, which might be varied due to the cell line specificity. All these data revealed the induction of calcium influx by THTMP, T0 and THTMP + T0 contributed the calcium mediated cell death of GBM cells.

The dissipation of mitochondrial membrane potential is an early indicator of apoptosis. It is also well known that mitochondria plays a key role in calcium homeostasis, and the rapid [Ca^2+^]i elevation leads to calcium overloading, and thus damages the mitochondrial membrane. Subsequently, apoptogenic factors released due to this damage also triggers the intrinsic apoptotic pathway [32,33,34]. Thus, to examine the effect of the compounds on the mitochondrial integrity, JC-1 assay was performed to evaluate the change in mitochondrial membrane potential (MMP). As the time increases, there was a significant loss in the MMP upon treatment with THTMP + T0 and THTMP than T0 and DMSO treated GBM cells. The intensity of the green fluorescence represents the cells losing its MMP which was directly correlated with the cell death or unhealthy cells, whereas the red fluorescence represents the healthy cells (Figure 6C). It was also evident that T0 treatment increased MMP over 24 h of treatment than the THTMP and THTMP + T0, which was correlated with the observation as less calcium influx on T0 treated GBM cells (Figure 6D). These experiments prove that the combinatorial drug was effective in inducing mitochondria-dependent pathway of apoptosis through calcium signaling.

To further explore the impact of combinatorial treatment induced apoptosis, we examined the fold change of ROS. As illustrated in Figure 6E, the compounds significantly induced ROS production in both GBM cells than the DMSO control. The combinatorial treatment increased the production of ROS with 3.5-fold change in MMK1 and approximately 3.0-fold in JK2 cells. This result agreed with the previous data, that the overexpression of GPR17 stimulates apoptosis by inducing ROS production and thereby inhibits glioma cell proliferation [35]. This data also suggested that THTMP + T0 was more efficient in inducing ROS-mediated apoptosis.

### 3.7. Combination of THTMP/T0 Induces Intrinsic Apoptotic Pathway Rather Than Extrinsic Apoptotic Pathway in GBM Cells

**Figure 7:**
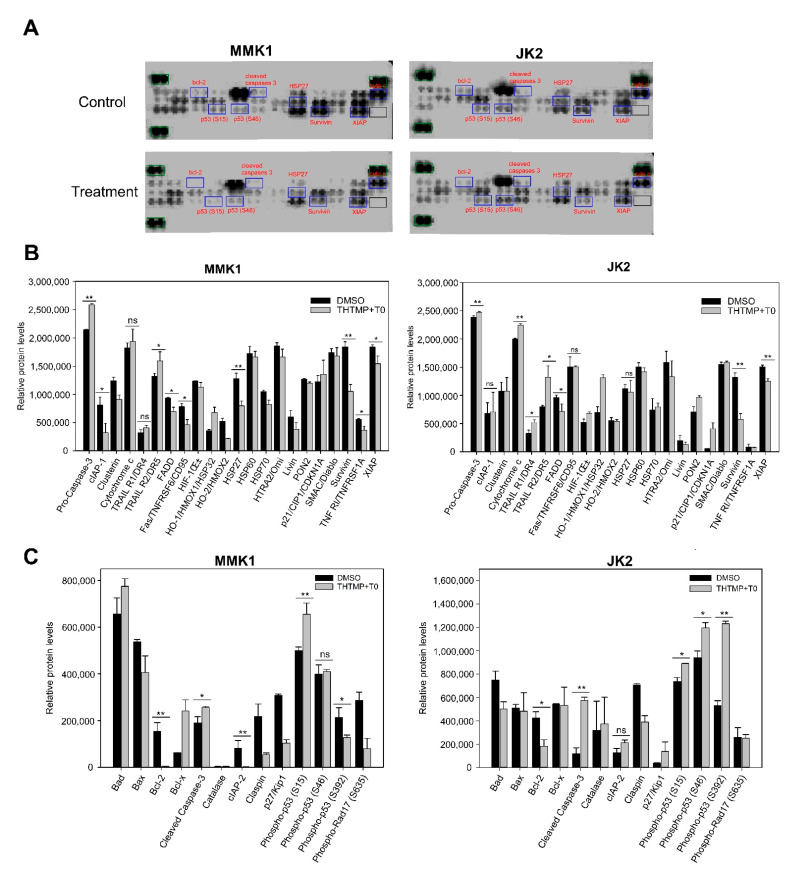
Proteome profiling of apoptosis-associated proteins. (**A**) Array images showing the expression of total 35 apoptosis-associated proteins upon combinatorial treatment with DMSO as control. (**B**) Relative level of expression of total 22 apoptotic proteins in the cell lysate of control and combinatorial treatment of MMK1 and JK2 cells (**C**) Integrated pixel intensity of 13 apoptotic proteins. The data were shown as mean ± SD; ns: not significant * indicates *p* < 0.05, ** *p* < 0.01.

Proteomic analysis was performed in both GBM cells to identify the potentially altered proteins associated with the intrinsic and extrinsic pathways of apoptosis. Human apoptosis proteome array was used, which includes 35 apoptosis-related proteins (Figure 7A). ImageJ was used to analyze apoptotic proteomic array to extrapolate the weak and strong signals from the images. The relative expression was quantified using the ratiometric analysis of the signals received from the sample and control. Various intrinsic proteins such as Bcl-2, cleaved caspases-3, cytochrome c, HSP27, cIAP-1, cIAP-2, p53, and XIAP were regulated upon combinatorial drug treatment in both GBM cells. The expression of Bcl-2, an anti-apoptotic protein family that regulates the mitochondria mediated apoptosis, was significantly reduced in both cell lines. In addition, the protein expression of cleaved caspase-3 and cytochrome c was found to be increased which deregulated the HSP27 (cytochrome c inhibitor) and Survivin (caspases 9 inhibitor) in both cell lines. We also noticed an increase in the expression of phospho-p53 (S15 and S46) in both the cells, which plays a key role in modulating its expression to induce apoptosis. We could observe a specific downregulation of cellular inhibitor of apoptosis proteins, cIAP-1 and cIAP-2 only in MMK1 cells, which remained unchangeable in JK2 cells. In addition, the level of XIAP (X-linked Inhibitor of Apoptosis Protein), which was highly correlated with a poor prognosis, significantly decreased upon combinatorial treatment than the control. Phospho p53 protein expression was found to be significantly increased in the treated cells, which plays a key role in both intrinsic and extrinsic apoptotic pathway. Other extrinsic proteins like the upregulation of death receptors, TNFR, TRAIL R1, TRAIL R2, FADD and Fas was observed, of which TNFR and TRAIL 1 function as proinflammatory receptors, while the remaining principally activate cell death pathways (Figure 7B,C).

### 3.8. In Vivo Anti-Tumor Efficacy of Combinatorial Drug, THTMP + T0

**Figure 8:**
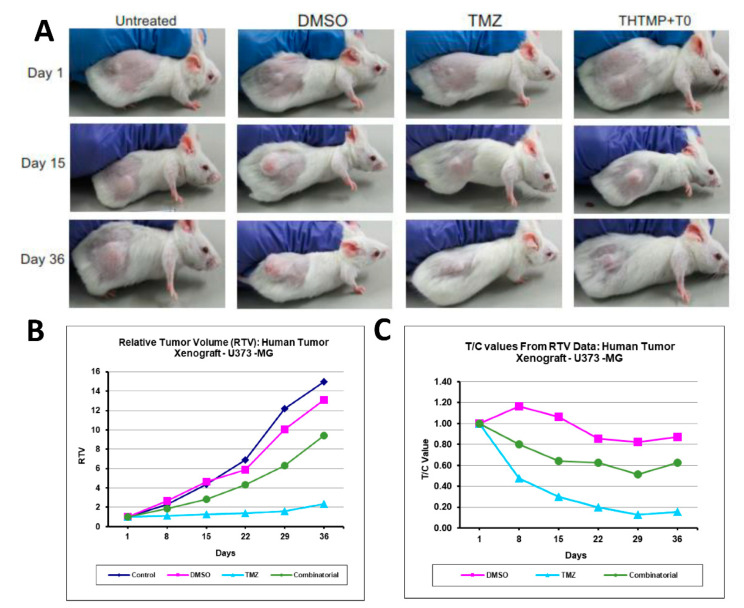
The anti-tumor efficacy of combinatorial treatment on glioblastoma xenograft model images of xenograft mice up to 36 days of treatment (**A**). Tumor growth curves of combinatorial treatment with TMZ as the positive control (**B**). The percentage treatment/control (T/C%) values or percent tumor regression values of combinatorial treatment and TMZ (**C**). No significant body weight change was observed after the drug treatment. RTV changes after the combinatorial treatment with *p*-value < 0.05 representing significant differences compared to vehicle control (DMSO) and positive control (TMZ).

In-vivo anti-tumor efficacy of combinatorial drug, THTMP + T0 was evaluated in a xenograft animal model (Figure 8A). In-vivo anti-cancer activity was evaluated against glioblastoma U373-MG Uppsala human tumor xenograft mice model. We used U373-MG Uppsala cell line, since it was challenging to develop a PDX model with RN1, PB1, MMK1, JK2 cell lines. The U373-MG Uppsala cell line was considered as the suitable cell line for developing tumor xenograft mice model, since it carries the same characteristics features of cell lines tested in vitro for high-grade human glioblastoma and confirmed the clinical correlation for testing drug. In addition, U373-MG Uppsala cell line derived animal model is the most frequently used model for testing the drug TMZ, an anti-GBM drug. Figure 8B,C revealed the relative tumor volume (RTV) and the relative activity criteria (T/C) of the combinatorial drug which was notably decreased when compared to the untreated and the DMSO control. Of note, less toxicity for the combinatorial drug was observed than the TMZ after the 8th day of treatment with similar trend effect throughout the experimental period of 36 days. Figure 8C revealed that the relative toxicity value decreased from 0.80 for combinatorial drug to 0.48 for TMZ treatment. This suggests that the TMZ has higher toxicity than the combinatorial treatment. The combinatorial drug has shown a time-sensitive reaction that was able to reduce the tumor volume with least toxicity compared to TMZ, thus it can prevent the GBM disease progression free survival through the targeted therapy. We also noticed the animal body weight was maintained in all conditions indicating non-systemic toxicity of the compounds (Supplementary Material).

## 4. Discussion

Currently TMZ is considered as a promising chemotherapeutic agent that significantly prolongs the survival of GBM patients. However, its clinical applicability was greatly reduced due to the resistance developed by MGMT expression and heterogeneity of GBM leading to treatment failure [36]. Previously, we characterized the phenolic compound, THTMP as an apoptosis inducer that significantly inhibited the GBM proliferation [14,37]. In addition, it was evident from the in-silico and in-vitro analysis, T0, a GPR17 agonist, strongly binds with GPR17 receptor and acts as a potent anticancer agent [16]. Thus, the approach of combining these two drugs along with TMZ could possibly favor the therapeutic effect against GBM treatment.

In the present study, we evaluated the cytotoxicity effect of THTMP, T0 and TMZ either as an individual drug and/or as combinatorial drug, against the GBM cells. The cells were found to be sensitive to THTMP and developed resistance to TMZ, which might be due to the unmethylation of the MGMT promoter of the GBM cells leading to the resistance to the alkylating chemotherapy agent, TMZ [28]. Despite the unmethylation of the cells, the combinatorial drug THTMP + TMZ has shown improved synergistic effect against GBM cells, with an additive effect for THTMP + T0 combination.

Our study not only demonstrated the anticancer property of the combinatorial treatment but also shed light on its possible molecular signaling pathway against GBM cells. The proliferation of cancerous cells occurs due to the absence of activated apoptotic signaling pathway [38]. The combinatorial treatment leads to the programmed cell death against GBM cells by activating various apoptotic factors, and increased cAMP level, an inhibitor of cell cycle progression and apoptosis inducer in cancer cells [39].

Moreover, various key parameters, like increased calcium, decreased MMP and increased ROS activate the intrinsic apoptotic signaling pathway upon combinatorial treatment. Intrinsic stimuli such as the accumulation of apoptotic mediators within the cell, in turn promotes Bax-induced cytochrome c release leading to the downstream activation of caspase 3 and 7 [40,41]. Survivin, a member of the apoptosis protein inhibitor, inhibits the activation of caspase 3 and 7 and thus blocks the cell death in most of the cancers [42]. The protein array data revealed that the upregulation of cleaved caspase-3 and cytochrome c with the downregulation of survivin [43] activates the intrinsic apoptotic pathway in GBM cells. Phosphor-p53, a tumor suppressor protein found to be upregulated, could control a wide number of genes involved in cellular processes including cell cycle arrest, cell senescence, DNA repair, metabolic adaptation and cell death. Induction of apoptotic death in nascent neoplastic cells was viewed as the primary mechanism by which p53 prevents tumor development [44]. Thus, p53 being both intrinsic and extrinsic apoptotic protein induces cell death and prevents the advancing of the disease.

The preclinical validation of the combinatorial drug in the in-vivo animal model also confirmed its promising anti-tumor role against GBM. THTMP + T0 was able to inhibit the GBM progression in-vivo that attributes for the prolonged survival. However, the inhibitory effect on GBM progression was not as robust as TMZ with the standard glioma cell lines, U373 Uppsala, which was used for xenograft animal model. TMZ exerts resistance to the prolonged therapy with hematological toxicity [45], oral ulceration, hepatotoxicity [46] and pneumocystis pneumonia [47] leading to discontinued therapy. Overall, the THTMP + T0 exerts less cytotoxicity at reduced concentration and could help in preventing the progression of the GBM disease through targeted therapy. The investigation on the combination of THTMP and T0 as GBM-selective anticancer agent in subcutaneous xenograft model explored the therapeutic potential of the drug. However, the ability of the combinatorial drug in crossing the blood–brain barrier needs further validation in GBM animal models. In addition, analyzing the in vitro and in vivo pharmacokinetics profiling might reveal the toxicity risk of the drug and thereby render its potentiality for the development of anti-GBM drug.

## 5. Conclusions

Our findings demonstrate that THTMP + T0 have a strong inhibitory effect on multiple GBM cells derived from GBM patient samples. THTMP also has a synergism effect with TMZ and T0, against GBM cell lines. Our study indicates that the combination of THTMP + T0 possesses better cytotoxicity effect in comparison to single THTMP or T0 or TMZ treatment on mesenchymal GBM cells. Moreover, the combinatorial treatment explores its ability to reduce migration, invasion, and colony formation of GBM cells and arrest the cell cycle at S phase. The mechanism of action of combinatorial drug against GBM cells occurs through the activation of mitochondrial-mediated apoptosis. In addition, the combinatorial treatment has a promising anti-tumor efficacy in GBM xenograft model, thereby possibly reducing the disease progression. Thus, we conclude that combination treatment of THTMP + T0 might be a promising candidate for the GBM drug development.

## Data Availability

The data are available from the corresponding authors upon request.

## Supplementary Materials

The following are available online at https://www.mdpi.com/article/10.3390/cells10081975/s1, Figure S1: (A) Survival data: Human tumor xenograft-U373-MG, (B) Animal body weight (grams) data: human tumor xenograft-U373-MG.
