# Supplementary figures and images for: Alkylaminophenol and GPR17 Agonist for Glioblastoma Therapy: A Combinational Approach for Enhanced Cell Death Activity
Source: Cells. 2021 Aug 3;10(8):1975. doi: 10.3390/cells10081975 (PMC8393831; doi:10.3390/cells10081975)

**A**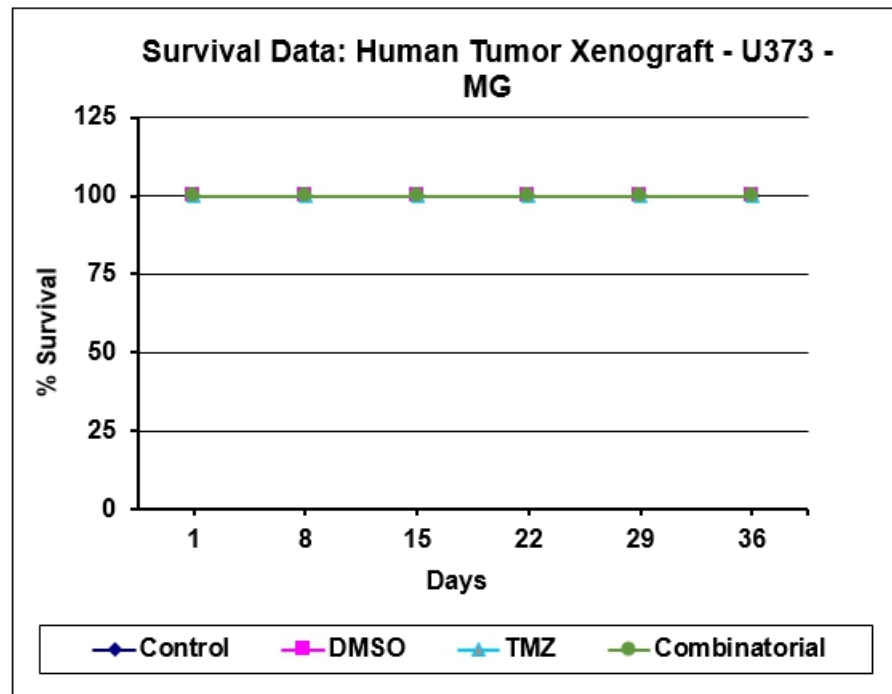**B**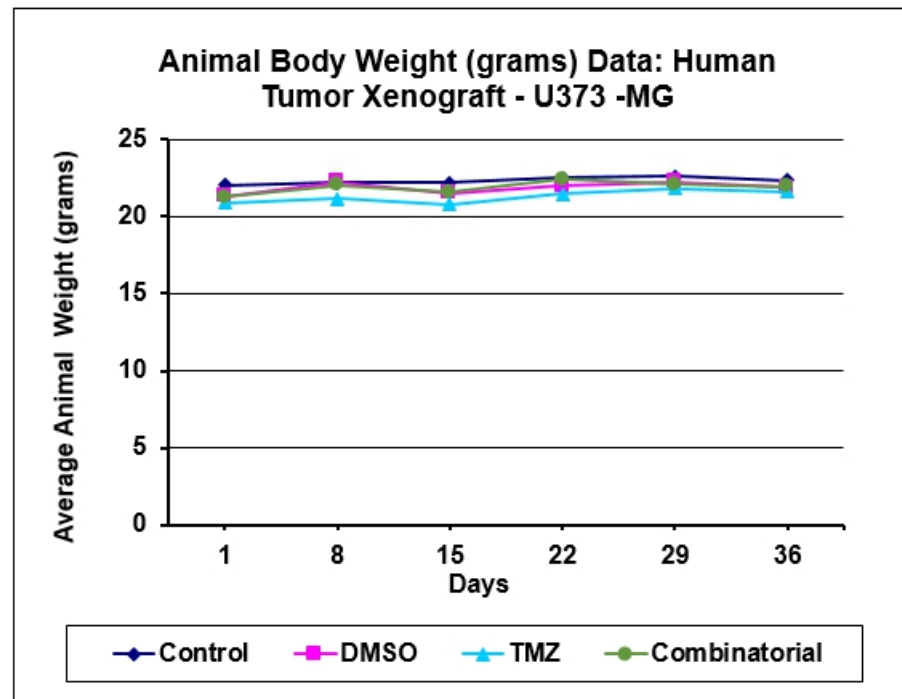

Supplement: Supplementary file 1 [file cells-10-01975-s001.zip › cells-1306053-supplementary.pdf]
